# Burden of disease from *Helicobacter pylori* infection in western Canadian Arctic communities

**DOI:** 10.1186/s12889-019-7065-x

**Published:** 2019-06-11

**Authors:** Katharine Fagan-Garcia, Janis Geary, Hsiu-Ju Chang, Laura McAlpine, Emily Walker, Amy Colquhoun, Sander Veldhuyzen van Zanten, Safwat Girgis, Billy Archie, Brendan Hanley, Andre Corriveau, John Morse, Rachel Munday, Karen J. Goodman, CANHelp Working Group

**Affiliations:** 1 Department of Medicine, Division of Gastroenterology, University of Alberta, Edmonton, Alberta Canada; 2 School of Public Health, University of Alberta, Edmonton, Alberta Canada; 3 Department of Laboratory Medicine and Pathology, University of Alberta, Edmonton, Alberta Canada; 4 Aklavik Health Committee, Aklavik, Northwest Territories Canada; 5 Government of Yukon, Whitehorse, Yukon Canada; 6 Government of the Northwest Territories, Yellowknife, Northwest Territories Canada; 7 Stanton Territorial Hospital, Yellowknife, Northwest Territories Canada; 8 Susie Husky Health Centre, Aklavik, Northwest Territories Canada

**Keywords:** *Helicobacter pylori*, Arctic, Canada, Indigenous health, Prevalence, Gastritis, Gastric cancer, Peptic ulcer disease

## Abstract

**Background:**

Indigenous communities across the circumpolar north have elevated *H. pylori* (*Hp*) prevalence and stomach cancer incidence. We aimed to describe the *Hp*-associated disease burden among western Canadian Arctic participants in community-driven projects that address concerns about health risks from *Hp* infection.

**Methods:**

During 2008–2013, participants underwent *Hp* screening by urea breath test and gastroscopy with gastric biopsies. We estimated *Hp* prevalence and prevalence by *Hp* status of endoscopic and histopathologic diagnoses.

**Results:**

Among 878 participants with *Hp* status data, *Hp* prevalence was: 62% overall; 66% in 740 Indigenous participants; 22% in 77 non-Indigenous participants (61 participants did not disclose ethnicity); 45% at 0–14 years old, 69% at 15–34 years old, and 61% at 35–96 years old. Among 309 participants examined endoscopically, visible mucosal lesions were more frequent in the stomach than the duodenum: the gastric to duodenal ratio was 2 for inflammation, 8 for erosions, and 3 for ulcers. Pathological examination in 308 participants with gastric biopsies revealed normal gastric mucosa in 1 of 224 *Hp*-positive participants and 77% (65/84) of *Hp*-negative participants with sharp contrasts in the prevalence of abnormalities between *Hp*-positive and *Hp*-negative participants, respectively: moderate-severe active gastritis in 50 and 0%; moderate-severe chronic gastritis in 91 and 1%; atrophic gastritis in 43 and 0%; intestinal metaplasia in 17 and 5%.

**Conclusions:**

The observed pattern of disease is consistent with increased risk of stomach cancer and reflects substantial inequity in the *Hp*-associated disease burden in western Arctic Canadian hamlets relative to most North American settings. This research adds to evidence that demonstrates the need for interventions aimed at reducing health risks from *Hp* infection in Indigenous Arctic communities.

## Background

Indigenous Peoples residing in northern Canada have an elevated prevalence of *Helicobacter pylori* (*Hp*) infection relative to southern Canadians [1]. This bacterial infection can persist lifelong, causing chronic inflammation of the stomach lining; in a small proportion of cases (5–15%), it causes peptic ulcer disease and, more rarely, stomach cancer [2]. Elevated *Hp* prevalence and stomach cancer rates have been observed in Indigenous communities across the circumpolar north relative to the average occurrence in the respective countries [1].

Systematic reviews of population-based studies report *Hp* prevalence estimates over 50% across most of Africa, Asia and Latin America, with lower and declining prevalence in Australia-New Zealand, Europe and North America [3, 4]. While *Hp* prevalence varies substantially within countries by ethnicity and socioeconomic status, and many region-specific prevalence estimates come from unrepresentative samples [5], rough regional estimates range from 24% for Australia-New Zealand to 79% for Africa, with prevalence in Canada and the United States estimated at 36 and 38%, respectively, in a 2017 systematic review [3]. Evidence from the late twentieth century showed *Hp* prevalence inversely associated with socioeconomic status within Europe and the United States [6, 7]. Because the infection typically is acquired in childhood, observed increases in *Hp* prevalence with age result from a cohort effect reflecting transmission levels within the first years after birth. Decreases in *Hp* prevalence observed in younger age groups in affluent countries suggests that transmission is decreasing in such countries, though it remains high in socioeconomically disadvantaged groups. In Canada, for example, the prevalence in pediatric patients residing in major urban centers was estimated in 2005 at just 5%, while 56% (92/163) of Wasagamack Cree children in northern Manitoba screened positive for *Hp* in 2002 [2, 8].

The Canadian North *Helicobacter pylori* (CAN*Help*) Working Group, a collaboration of academic scientists with Indigenous community leaders and their health care providers [9], conducts community-driven investigations focused on *Hp* infection in the Northwest Territories (NT) and Yukon (YT) [10–16]. Incorporating the perspective of those who bear the burden, results from these projects will be used to develop *Hp* control strategies that are cost-effective and culturally appropriate for Arctic Indigenous communities. Previous reports describe details of CAN*Help* projects and their community-driven approaches [10, 12–15, 17]. This paper describes the burden of disease from *Hp* infection among participants in CAN*Help* community projects.

## Methods

### Research sought by communities

The CAN*Help* research program arose from the confluence of three constituencies: residents of western Canadian Arctic communities worried about *Hp* infection and its link to stomach cancer; health care practitioners frustrated by poor effectiveness of available clinical management strategies for this frequently encountered infection; and public health officials seeking evidence to inform infection control strategies. In the early 2000s, NT health care officials sought input from University of Alberta researchers to respond to concerns voiced by community leaders. In 2006, a meeting between academic scientists and NT medical directors generated support for community-driven research aimed at describing the burden of disease from *Hp* infection in concerned communities. NT health care partners recommended the Hamlet of Aklavik for the initial project because Aklavik community leaders had advocated for research to reduce health risks from *Hp* infection. The Aklavik *H. pylori* Project launched in 2007. Word of its success generated interest in neighboring communities. Invited by community leaders, the research team launched community *H. pylori* Projects in Old Crow YT in 2010, Tuktoyaktuk NT in 2011 and Fort McPherson NT in 2012. Projects launched in 2016–2017 are not included in this report. The University of Alberta Health Research Ethics Board approved this research, and as required by law, we obtained annual research licenses in both NT and YT before collecting data.

### Participating communities

**Fig. 1:**
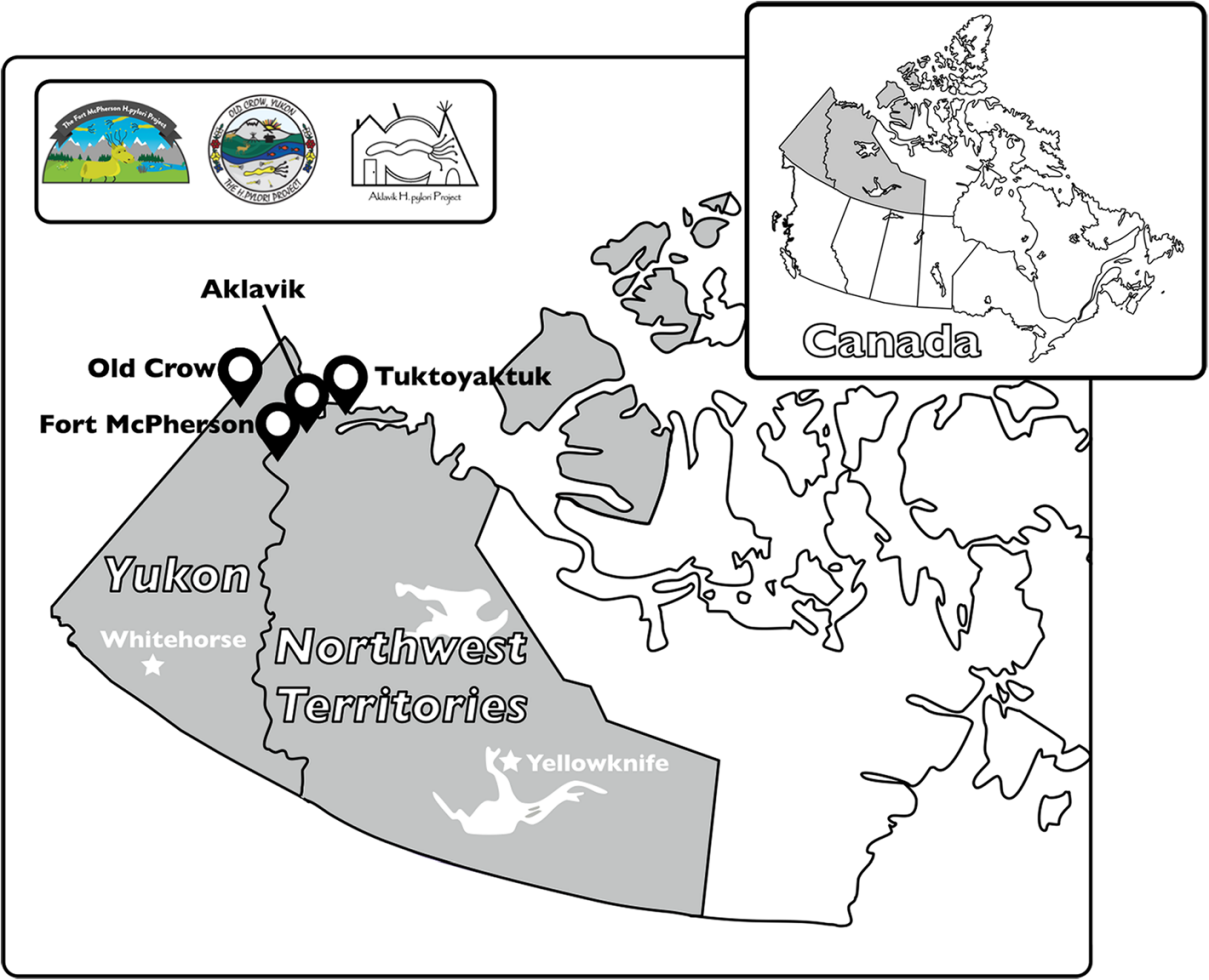
Map of the western Canadian Arctic with partner community locations indicated. Inset shows logos of community projects that had contests to select a logo created by a local artist: Fort McPherson *H. pylori* Project (local artist – Johanna Edwards); Old Crow *H. pylori* Project (local artist – Mary Jane Moses); Aklavik *H. pylori* Project (local artist – Richard Papik). Map adapted from https://commons.wikimedia.org/wiki/File:Map_Canada_political-geo.png

Population estimates (from census nearest project launch) were 594 (2006) for Aklavik, 245 (2011) for Old Crow, 854 (2011) for Tuktoyaktuk, and 792 (2011) for Fort McPherson (Fig. 1) [18–21]. Most residents of participating communities identify as Indigenous: by census counts, in Aklavik, 92% were Indigenous (mainly Inuvialuit (western Canadian Inuit) or Gwich’in (Athabaskan) First Nation); in Old Crow, 90% were Vuntut Gwitchin (Athabaskan) First Nation; in Tuktoyaktuk, 92% were Inuvialuit; and in Fort McPherson, 94% were Indigenous (mainly Gwich’in) [18, 22]. Aklavik, 113 km south of the Arctic Coast, is accessible by air, ice road in winter and water in summer [23]. Old Crow, north of the Arctic Circle on the Porcupine River, is accessible only by air [24]. Tuktoyaktuk, on the Arctic Ocean coast, is accessible by air, highway year-round since November 2017 (before that by ice road in winter). Fort McPherson, on the Peel River, is accessible by highway year-round [23].

### Community-driven approach

Each project launch required the formation of a planning committee of community residents; the process for recruiting committee members was devised by community partners. An exception was the Tuktoyaktuk project, launched at the request of the Inuvialuit Regional Corporation [25], with planning occurring in partnership with regional Inuvialuit leadership and a Tuktoyaktuk community health representative. Planning committees and other community partners provided input on design and implementation of all research activities to ensure they adhered to community priorities and cultural values. Planning committees and other community partners reviewed all research reports before they were made public.

### Study population and design

Project recruitment activities in each community encouraged all residents to participate in baseline research activities: interviewer-administered questionnaires; *Hp* screening; and endoscopy with gastric biopsies for culture and pathological examination. After endoscopy activities concluded, randomized trials offered treatment to eliminate *Hp* infection to those testing positive. Participants gave written informed consent for overall project participation with additional consents for endoscopy and treatment. Participants < 17 years old required written parental consent, with children deemed sufficiently mature providing written assent. Details can be found on the CAN*Help* Working Group website [9].

### *Hp* screening

Beginning in January 2008, we offered screening for *Hp* infection using the 13C-urea breath test (UBT), the most accurate non-invasive test for detecting *Hp* infection in children and adults [26]; most well-designed validation studies estimate both sensitivity and specificity greater than 95% against biopsy-based diagnosis [27], though it should be noted that biopsy-based diagnosis is not an optimal gold standard because tissue sampling can miss *Hp* in the stomach due to its patchy distribution and diagnostic accuracy also depends on tissue preparation techniques and observer expertise [26, 28, 29]. The UBT avoids these limitations, but its accuracy depends on optimal breath sample collection [30]. We used nondispersive isotope-selective infrared spectroscopy to measure the 13C/12C ratio in breath samples collected before and after consumption of 13C-labeled urea [31, 32]; a positive result reflects presence of urease in the stomach, a highly specific marker for *Hp* infection. We asked participants to avoid acid-suppressing medications for 24 h before the test and to fast except for water for 4 h before the test. We obtained a baseline breath sample, asked participants to swallow 50–75 mg of 13C-labeled urea dissolved in citric acid, and collected a second breath sample 30 min after the urea solution was swallowed [17].

### Upper gastrointestinal endoscopy, histopathology, and microbiological culture

Using ultra-slim Olympus gastroscopes, gastroenterologists performed unsedated upper gastrointestinal endoscopy for consenting participants, regardless of *Hp* status, in temporary endoscopy clinics organized in community health centers (2008 in Aklavik, 2012 in Old Crow, 2013 in Fort McPherson and at the Inuvik Regional Hospital for Tuktoyaktuk participants) [33]. The regional planning approach used for the Tuktoyaktuk project created logistic constraints that reduced time periods, relative to the other community projects, for recruiting participants and carrying out project activities; as a result, participation was not sufficient to warrant resources required for an endoscopy team to visit this community. Instead, we offered to pay travel expenses for Tuktoyaktuk residents willing to undergo endoscopy in Inuvik (a 2-h drive). Gastric biopsies collected during endoscopy were transported to the University of Alberta for histopathologic examination (2–3 from antrum, 2–3 from corpus) and tissue culture (1 from antrum, 1 from corpus) [14]. A single pathologist (SG) evaluated antral and corpus biopsies separately, grading *Hp* density, active and chronic gastritis, gastric atrophy, and other neoplastic lesions using the updated Sydney system [34]. We differentiate active gastritis, characterized by polymorphonuclear neutrophil infiltration occurring in the context of chronic inflammation, from chronic gastritis, characterized by the presence of mononuclear cells, chiefly lymphocytes, plasma cells and macrophages [34]. For culture, antral and corpus biopsies were pooled and mechanically homogenized; resulting suspensions were plated on brain heart infusion/yeast extract/5% horse serum agar plates and incubated at 37 °C in microaerophilic conditions. Cultures were checked for growth every 48 h, for up to a month. Colonies consistent with *Hp* growth were sub-plated and expanded for generation of glycerol stocks. *Hp* classification was confirmed by urease, catalase, oxidase and 16S ribosomal RNA PCR testing.

### *Hp* infection status

We classified *Hp* infection status using all available results (UBT, histopathologic examination, and/or bacterial culture) for each participant. When results were discordant, we used an algorithm based on the probability that the status was negative or positive (for example, UBT positive + histopathology positive + culture negative was classified as positive; UBT negative + histopathology positive + culture negative was classified as negative). Among 254 participants with classifiable *Hp* status from 3 tests, 216 (85%) were concordant on all 3 tests; among 53 participants with classifiable *Hp* status from just 2 tests, 47 (89%) had concordant status. Of the 3 tests, UBT and histopathology agreed most frequently, for 263 of 272 (97%) participants with results from both, while the results of culture diverged more frequently, agreeing with UBT for 218 of 254 (86%) participants with both of these tests and with histopathology for 252 of 289 (87%) participants with both of these tests. The more frequent discordance of culture is not surprising given the technical challenges inherent in tissue culture of *Hp* in the laboratory [30, 35]. The high agreement of UBT and histopathology indicates comparable and excellent accuracy of these 2 diagnostic tests. Comparing UBT against histopathology as the gold standard, estimated sensitivity is 96% (95% confidence interval (CI), 94–99%) and estimated specificity is 97% (95% CI, 94–100%). Conversely, comparing histopathology against UBT as the gold standard, estimated sensitivity is 99% (95% CI, 98–100%) and estimated specificity is 91% (95% CI, 85–97%).

### Statistical analysis

The cross-sectional design is appropriate for investigations of the burden of disease from *Hp* in a community setting, given that the infection onset generally goes undetected, the infection often persists indefinitely without symptoms, and gastric disease caused by the infection is often asymptomatic. Screening for prevalent cases is, therefore, the starting point for describing the occurrence of *Hp* infection and related disease in a population. To describe the burden of infection in participating communities, we present the estimated prevalence of *Hp* infection and 95% confidence intervals by age, sex, ethnicity and community. To describe the burden of gastric disease related to *Hp* infection and demonstrate relatedness of specific conditions to *Hp* infection, we present the estimated prevalence of endoscopic diagnoses (esophagitis/esophageal erosions, Barrett’s esophagus, gastritis, gastric erosions, gastric ulcer, duodenitis, duodenal erosions, duodenal ulcer) and histopathologic diagnoses (active gastritis, chronic gastritis, gastric atrophy, benign MALT hyperplasia or lymphoid aggregates, lymphoepithelial lesions, intestinal metaplasia, dysplasia, carcinoma) and 95% CIs by *Hp* status. In analyses of endoscopy or histopathology results, we excluded the small number of participants who underwent treatment to eliminate *Hp* after the screening UBT and before endoscopy.

## Results

### Participation and data availability

The four community projects enrolled 934 participants, representing 38% of the combined populations of these communities (Table 1). The first two community projects had the highest participation, with 64 and 85% of residents of Aklavik and Old Crow, respectively. *Hp* status was available for 878 (94%) of the 934 participants. Table 2 shows participation in UBT screening and endoscopy by community, with UBT results available for 90% (841/934) and histopathology results available for 33% (308/934). Due to the logistic challenges, only 5 Tuktoyaktuk residents had eligible histopathology results: each fell in a different age group; 4 were women; 5 were Indigenous (4 were Inuvialuit, the dominant ethnicity in Tuktoyaktuk); and 4 were *Hp*-positive with a distribution of endoscopic and histopathologic outcomes reflective of the larger study population.

**Table 1:** Participation in CAN*Help* projects and availability of *Hp* status data, western Arctic Canadian communities, 2007–2013

Community	Launch Year	Census-estimated^a^ Population	Enrolled Participants (signed consent)		Participants with Data on *H. pylori* Status	
			n	% of Census	n	% of Participants
Aklavik	2007	594	383	64	352	92
Old Crow	2010	245	208	85	200	96
Tuktoyaktuk	2011	854	107	13	102	95
Fort McPherson	2012	792	236	30	224	95
Total		2485	934	38	878	94

^a^From 2006 census for Aklavik [18]; from 2011 census for other communities [19–21]

**Table 2:** Participation in CAN*Help* project diagnostic testing and availability of results, western Arctic Canadian communities, 2008–2013

Community	Enrolled Participants	UBT				Endoscopy			
		Completed		Classifiable Result		Completed		Histopathology Results	
	n	n	%	n	%	n	%	n	%
Aklavik	383	334	87	333	87	196	51	194	51
Old Crow	208	199	96	192	92	59	28	59	28
Tuktoyaktuk	107	104	97	102	95	5	5	5	5
Fort McPherson	236	228	97	214	91	50	21	50	21
Total	934	865	93	841	90	310	33	308	33

Table 3 shows the distribution of age, sex, community and ethnicity in all 934 participants and in two study population subsets: the 878 with *Hp* status and the 308 with histopathology results. Aside from the exclusion of young children from endoscopy, the age distribution of the two subpopulations approximates that of the total study population. The sex distribution is similar across study population subsets, with women accounting for 54–56% of each. The proportion identifying as Indigenous is 90–92% across study subpopulations. The representation of communities is nearly identical in the total study population and among those with *Hp* status, but among those with histopathology results Aklavik is overrepresented due to a much higher proportion of participants undergoing endoscopy in the first project, and Tuktoyaktuk is underrepresented due to only 13 participants traveling to Inuvik for endoscopy. Just 56 (6%) participants lacked data on *Hp* status; these participants were disproportionately younger and male.

**Table 3:** Characteristics of CAN*Help* project participants by data availability, 2007–2013

	All 934 Participants		878 Participants with *Hp* Status		308 Participants with Histopathology		56 Participants without *Hp* Status	
	n	%^a^	n	%^a^	n	%^a^	n	%^a^
Age	χ^2^-*p*-value^b^: 0.006							
0–9.75	76	8	65	7	0	0	11	20
9.8–14^c^	45	5	41	5	8	3	4	7
15–24	136	15	124	14	46	15	12	22
25–34	142	15	135	15	45	15	7	13
35–44	121	13	118	13	41	13	3	6
45–54	170	18	165	19	75	24	5	9
55–64	133	14	128	15	57	18	5	9
65–96	109	12	102	12	36	12	7	13
*missing*	*2*		*0*		*0*		*2*	
Sex	χ^2^-*p*-value^b^: 0.09							
Male	430	46	398	45	137	44	32	57
Female	504	54	480	55	171	56	24	43
*missing*	*0*		*0*		*0*		*0*	
Community	χ^2^-*p*-value^b^: 0.15							
Aklavik	383	41	352	40	194	63	31	55
Old Crow	208	22	200	23	59	19	8	14
Tuktoyaktuk	107	11	102	12	5	2	5	9
Fort McPherson	236	25	224	26	50	16	12	21
*missing*	*0*		*0*		*0*		*0*	
Ethnicity	χ^2^-*p*-value^b^: 0.41							
Non-Indigenous	82	10	77	9	22	8	5	13
Indigenous	775	90	740	91	271	92	35	88
Inuvialuit (Inuit)	305	36	289	35	121	41	16	40
Gwich’in (First Nations)	424	49	405	50	133	45	19	48
Other/mixed^d^	46	5	46	6	17	6	0	0
*missing*	*77*		*61*		*15*		*16*	

*Hp* H. pylori

^a^% of column total minus missing; distributions do not all sum 100% due to rounding

^b^For the comparison of the distribution in participant groups with and without *Hp* status

^c^The 9.8–14 category includes two participants < 10: one who was 9 years and 10 months at the time of UBT and 9 years and 11 months at endoscopy, and one who was 9 years and 11 months at the time of UBT with no endoscopy

^d^Includes: Métis; mixed Indigenous ethnicities; unspecified Indigenous ethnicity; Indigenous mixed with non-Indigenous ethnicities

### *Hp* prevalence

Among 878 participants with *Hp* status, 62% were *Hp*-positive (Table 4). The prevalence was 45% (95% CI, 36–55%) in children under 15, 69% (95% CI, 63–74%) in the 15–34-year age group, and 61% (95% CI, 57–66%) in the 35–96-year age group. The prevalence was somewhat higher in men (65%; 95% CI, 60–70%) than women (59%; 95% CI, 55–63%). Across communities, the prevalence ranged from 57% in Tuktoyaktuk to 68% in Old Crow. The largest variation in prevalence was by ethnicity, with non-Indigenous participants having a much lower prevalence (22%; 95% CI, 13–31%) than Indigenous participants (66%; 95% CI, 62–69%).

**Table 4:** *Hp* prevalence by demographic characteristics, 878 CAN*Help* project participants with *Hp* status, 2008–2013

	n	*Hp* Prevalence		
		n	%	95% CI
Total	878	541	62	58, 65
Age				
0–9.75	65	30	46	34, 58
9.8–14^a^	41	18	44	29, 59
15–24	124	85	69	60, 77
25–34	135	93	69	61, 77
35–44	118	70	59	50, 68
45–54	165	103	62	55, 70
55–64	128	78	61	52, 69
65–96	102	64	63	53, 72
Sex				
Male	398	258	65	60, 70
Female	480	283	59	55, 63
Community				
Aklavik	352	213	61	55, 66
Old Crow	200	136	68	62, 74
Tuktoyaktuk	102	58	57	47, 66
Fort McPherson	224	134	60	53, 66
Ethnicity				
Non-Indigenous	77	17	22	13, 31
Indigenous	740	486	66	62, 69
Inuvialuit (Inuit)	289	193	67	61, 72
Gwich’in (First Nations)	405	260	64	60, 69
Other/mixed^b^	46	33	72	59, 85
*missing*	*61*			

*Hp* H. pylori, *CI* binomial Wald confidence interval

^a^The 9.8–14 category includes two participants < 10: one who was 9 years and 10 months at the time of UBT and 9 years and 11 months at endoscopy, and one who was 9 years and 11 months at the time of UBT with no endoscopy

^b^Category includes: Métis; mixed Indigenous ethnicities; unspecified Indigenous ethnicity; mixed Indigenous and non-Indigenous ethnicities

### Endoscopic assessment

Table 5 shows the prevalence of abnormalities observed during endoscopy by *Hp* status. Of note, 79% of *Hp*-positive participants had gastric mucosa that appeared normal. Visible esophagitis and Barrett’s esophagus were more prevalent in *Hp*-negative participants (11 and 7%, respectively) than *Hp*-positive participants (8 and 4%, respectively). Paradoxically, the prevalence of visible gastritis, gastric erosions and gastric ulcers was not markedly different in groups defined by *Hp* status: 12–15% had visible gastritis, 8–11% had gastric erosions, and 3–4% had gastric ulcers. The prevalence of visible duodenal lesions was lower: duodenal lesions were observed in 9% of *Hp*-positive participants and 4% of *Hp*-negative participants. No *Hp*-negative participants had visible duodenal erosions or ulcers and 4% had duodenitis, while 1% of *Hp*-positive participants had duodenal erosions, 1% had duodenal ulcers, and 7% had duodenitis. In all 308 participants with complete endoscopic assessment, the gastric to duodenal ratio was 2 for inflammation, 8 for erosions, and 3 for ulcers. In 271 Indigenous participants, the gastric to duodenal ratio was 2 for inflammation, 6 for erosions, and 8 for ulcers.

**Table 5:** Prevalence of endoscopic abnormalities by *Hp* status, 309 CAN*Help* project participants with endoscopy data, 2008–2013

	*Hp* positive			*Hp* negative		
	(*n* = 224)			(*n* = 85)		
	n	%	95% CI	n	%	95% CI
Normal gastric mucosa^a^	177	79	74, 84	65	77	68, 86
Normal duodenal mucosa	204	91	87, 95	82	96	93, 100
Normal gastric and duodenal mucosa^a^	162	72	66, 78	62	74	64, 83
Esophagitis/esophageal erosions	19	8	5, 12	9	11	5, 19
Barrett’s esophagus	8	4	2, 7	6	7	3, 15
Gastric inflammation, erosions, or ulcers^a^	46	21	15, 26	17	20	12, 29
Gastritis^a^	33	15	10, 19	10	12	5, 19
Gastric erosions^a^	17	8	4, 11	9	11	5, 19
Gastric ulcer^a^	6	3	1, 6	3	4	1, 10
Duodenal inflammation, erosions, or ulcers	20	9	5, 13	3	4	1, 10
Duodenitis	16	7	4, 11	3	4	1, 10
Duodenal erosions	3	1	0, 4	0	0	0, 4
Duodenal ulcer	3	1	0, 4	0	0	0, 4

*Hp* H. pylori, *CI* binomial Wald confidence interval (binomial exact CI for numerators < 10; one-sided binomial exact 97.5% CI for numerators of 0)

^a^One *Hp*-negative participant was missing gastric mucosa assessment, so the denominator is 84 for gastric abnormalities among *Hp*-negatives

We examined whether the similar frequencies of endoscopically observed gastric abnormalities in groups defined by *Hp* status were due to previous *Hp* infection eliminated by antimicrobial therapy among *Hp*-negatives or recent use of proton pump inhibitors (PPI) or H2-receptor antagonists (H2RA), which decrease the density of *Hp* organisms, thereby reducing the sensitivity of diagnostic tests. In the 84 *Hp*-negative participants with treatment history data, 30 (36%) had previous treatment; the proportion classified as having normal gastric mucosa was 80% among those treated previously and 77% among those not treated previously (for duodenal mucosa, these proportions were 97 and 96%, respectively). Among 302 participants with medication data, recent PPI/H2RA use was reported by 11% of *Hp*-positives and 27% of *Hp*-negatives. To assess the hypothesis that *Hp*-negatives with abnormal gastric mucosa were false negatives due to medication use, we compared the proportion classified as having normal gastric mucosa by PPI/H2RA use: in 85 *Hp*-negatives, 74% among users and 79% among non-users (for duodenal mucosa, 91 and 98%, respectively); in 218 *Hp*-positives, 68% among users and 80% among non-users (for duodenal mucosa, 96 and 91%, respectively). Thus, this paradox does not appear to be due to previously treated *Hp* infection or medication use among current *Hp*-negatives.

### Histopathologic assessment

Groups defined by *Hp* status had strikingly different frequencies of abnormal histopathology (Table 6). The proportion with normal gastric mucosa was 77% among *Hp*-negative participants evaluated and just 1 of 224 *Hp*-positive participants evaluated. Compared to *Hp*-negative participants, *Hp*-positive participants not only had a much higher prevalence of histologic abnormalities, but they also had a much higher severity gradient, although the frequency of intestinal metaplasia was low in both groups and the difference between groups less striking. Of the 4 *Hp*-negatives with intestinal metaplasia, 2 had a record of previous treatment to eliminate *Hp*.

**Table 6:** Severity of gastric pathology by *Hp* status, 308 CAN*Help* project participants with histopathology data, 2008–2013

Pathology	*Hp* positive (*n* = 224)			*Hp* negative (*n* = 84)			
	n	%^a^	95% CI	n	%^a^	95% CI	χ^2^-*p*-value^e^:
Normal histopathology^b^							
No	223	99.6	99, 100	19	23	14, 32	0.000
Yes	1	0.4	0, 2	65	77	68, 86	
Active gastritis^c^							
None	7	3	1, 6	82	98	94, 100	0.000
Mild	102	46	39, 52	2	2	0, 8	
Moderate	81	36	30, 42	0	0	0, 4	
Severe	32	14	10, 19	0	0	0, 4	
Chronic gastritis							
None	2	1	0, 2	73	87	80, 94	0.000
Mild	17	8	4, 11	10	12	5, 19	
Moderate	99	44	38, 51	1	1	0, 6	
Severe	106	47	41, 54	0	0	0, 4	
Atrophy							
None	128	57	51, 64	84	100	96, 100	0.000
Mild	68	30	24, 36	0	0	0, 4	
Moderate	23	10	6, 14	0	0	0, 4	
Severe	5	2	1, 5	0	0	0, 4	
Intestinal metaplasia							
None	185	83	78, 88	80	95	91, 100	0.002
Mild	23	10	6, 14	4	5	1, 12	
Moderate	13	6	3, 9	0	0	0, 4	
Severe	3	1	0, 4	0	0	0, 4	
MALT hyperplasia^d^ (*n* = 114; 96 HP+)							
None	3	3	1, 9	13	72	52, 93	0.000
Mild	34	35	26, 45	5	28	10, 53	
Moderate	45	47	37, 57	0	0	0, 19	
Severe	14	15	8, 22	0	0	0, 19	
Lymphoid aggregates^d^ (*n* = 194; 128 HP+)							
Present	74	58	49, 66	5	8	3, 17	0.000
Lymphoepithelial lesions							
Present	7	3	1, 6	0	0	0, 4	0.101
Dysplasia or Carcinoma							
Present	0	0	0, 2	0	0	0, 4	n/a

*Hp* H. pylori, *CI* binomial Wald confidence interval (binomial exact CI for numerators < 10; one-sided binomial exact 97.5% CI for numerators of 0)

^a^Distributions do not all sum 100% due to rounding

^b^Excludes MALT hyperplasia or lymphoid aggregates; includes reactive gastropathy

^c^2 *H. pylori*-positive participants lack classification for active gastritis

^d^In 2008, presence/absence of lymphoid aggregates was noted without grading MALT hyperplasia; after 2008, MALT hyperplasia was graded instead of noting presence/absence of lymphoid aggregates

^e^From score test for trend of odds

The results show good statistical precision for describing the severity distributions of the histopathological outcomes and differentiating these distributions in groups defined by *Hp* status. The study population yields even greater precision for differentiating the presence or absence of these outcomes: active gastritis occurred in 96% (95% CI, 92–98%) of positives and 2% (95% CI, 0–8%) of negatives; chronic gastritis occurred in 99% (95% CI, 98–100%) of positives and 13% (95% CI, 6–20%) of negatives; gastric atrophy occurred in 43% (95% CI, 36–49%) of positives and 0% (95% CI, 0–4%) of negatives; intestinal metaplasia occurred in 17% (95% CI, 12–22%) of positives and 5% (95% CI, 1–12%) of negatives; MALT hyperplasia or lymphoid aggregates occurred in 74% (95% CI, 69–80%) of positives and 12% (95% CI, 5–19%) of negatives; lymphoepithelial lesions occurred in 3% (95% CI, 1–6%) of positives and 0% (95% CI, 0–4%) of negatives. Thus, each of these histopathological outcomes was strongly associated with prevalent *Hp* infection in this population.

## Discussion

This analysis shows a high *Hp*-associated disease burden in four western Canadian Arctic hamlets, with an estimated *Hp* prevalence of 66% among Indigenous residents and 22% among non-Indigenous residents. Among participants examined endoscopically, visible inflammation, erosions and ulcers were more frequent in the stomach relative to the duodenum. Pathological examination revealed a low prevalence of mild abnormalities among *Hp*-negative participants; in contrast, *Hp*-positive participants had a high prevalence of moderate-severe active and chronic gastritis; the prevalence of atrophic gastritis was 43% among *Hp*-positive participants and 0 among *Hp*-negative participants, while the prevalence of intestinal metaplasia was 17% among *Hp*-positive participants and 5% among *Hp*-negative participants.

The pattern of disease observed in this population is consistent with increased risk of stomach cancer [36]. While no cases of dysplasia or carcinoma were detected, none would be expected due to small numbers. During 2008–2016, 3 of the 726 NT participants in this analysis had gastric cancer diagnosed and reported to the NT Cancer Registry (NT Department of Health and Human Services staff, personal communication, July 2018). While small community sizes preclude meaningful estimates of the frequency of gastric dysplasia or carcinoma, the combined community study population yields good statistical precision for estimating the prevalence and severity distributions of less advanced *Hp*-associated pathological outcomes. The elevated ratio of gastric to duodenal lesions is the inverse of the pattern observed in populations where the risk of stomach cancer is low and *Hp* infection frequently leads to duodenal ulcers [36–38]. The more frequent occurrence of gastric ulcer relative to duodenal ulcer has been observed in other populations at increased risk of stomach cancer [37, 38]. In addition, chronic gastritis and gastric atrophy are initial stages in Correa’s widely accepted model of gastric carcinogenesis [39]; thus, the high prevalence of these conditions are further indications of increased stomach cancer risk in participating communities.

The estimated prevalence of endoscopically and histopathologically diagnosed gastric disease associated with *Hp* infection in CAN*Help* community project participants is a rare resource given that very few community-based studies have assessed geographically-defined communities for these diagnoses. A recent clinic-based study of 432 Alaska Natives undergoing endoscopic assessment for digestive symptoms estimated similarly high prevalence of stomach pathology in *Hp*-positive participants [40]; prevalence estimates for Alaska Native patients and Indigenous CAN*Help* project participants, respectively, were 78 and 97% for active gastritis, 98 and 99% for chronic gastritis, and 13 and 17% for intestinal metaplasia [40]. Estimated prevalence of stomach pathology in *Hp*-negative participants was higher in Alaska Native patients than CAN*Help* project participants, respectively: 18 and 3% for active gastritis; 69 and 14% for chronic gastritis; and 10 and 6% for intestinal metaplasia [40]. The Alaska Native patients had much higher prevalence of endoscopically detectable gastric disease than the CAN*Help* project participants (for example, 85% of Alaska Native patients had an endoscopic diagnosis of gastritis, in contrast to 14% of CAN*Help* project participants), which is likely to be due, at least in part, to the clinic-based study design that recruited symptomatic patients who would have more severe disease on average than a community-based population [40]. In addition, the ratio of gastric to duodenal ulcers in the Alaska Native participants was nearly 5 (33:7) [40].

The pattern of gastric disease observed among *Hp*-positive CAN*Help* community project participants contrasts sharply with the pattern we reported previously for *Hp*-positive patients with gastric biopsies evaluated at the University of Alberta Hospital in Edmonton, Alberta (metro area 2016 census population = 1,321,426) between April 2010 and March 2011 [14, 41]. In this patient population (*Hp* prevalence = 14% in ~ 3000 patients assessed), of roughly 400 *Hp*-positive patients evaluated, 11% had active gastritis, 40% had mild chronic gastritis, 55% had moderate chronic gastritis, 5% had severe chronic gastritis, and just 2% had gastric atrophy. Thus, compared to an urban southwestern Canadian population of patients of predominantly European ancestry [41] evaluated for digestive complaints, the community-based study population of residents of predominantly Indigenous western Canadian Arctic hamlets had a prevalence of *Hp* infection over 4 times higher along with a notably more severe pattern of gastric mucosal injury among those infected. This contrast reveals substantial inequity in the disease burden associated with *Hp* infection in western Arctic Canadian hamlets relative to a North American metropolis.

In addition to the small study size for estimating rare outcomes, another limitation of this investigation is the possibility that the participants did not accurately represent the participating communities. Within the constraints of available resources, every effort was made to include all residents of participating communities. If those who did not participate included a greater proportion of community members who were less eager to engage in health care, then we would likely have underestimated the prevalence of *Hp* infection and associated pathology. There was also potential for misclassification of *Hp* status and histopathology outcomes given imperfect diagnostic methods. The accuracy estimates generated by our data for classifying *Hp* status, however, suggest that our *Hp* prevalence estimates are roughly accurate for the study population. Also, the comparison of our histopathology results to those of *Hp-*positive patients evaluated by the University of Alberta Hospital pathology laboratory, where gastric biopsies from our community projects were processed and assessed, enhances the validity of the distinct patterns observed in the Arctic communities.

## Conclusions

We have offered an example of the value of community-driven investigations for generating descriptions of the public health burden from diseases identified by affected communities and their healthcare providers to be of high community impact. This research provides evidence of a high burden of disease from *Hp* infection in Indigenous communities of western Arctic Canada. These results add to a small body of evidence that demonstrates the need for targeted interventions aimed at reducing health risks from *Hp* infection in Indigenous Arctic communities. In keeping with community-university research agreements, all results have been disseminated within participating communities to address voiced concerns and support community efforts to advocate for relevant resources and policies. In addition, our collaborative team has used these disease burden results, along with results from treatment trials and longitudinal follow-up [17, 42], to create *Hp* clinical guidelines (not yet published) specific to healthcare practitioners serving Arctic communities in Canada.

## Data Availability

The datasets used for the current study are available from the corresponding author on reasonable request following community review of proposed data uses.

## Abbreviations

CAN*Help*: Canadian North *Helicobacter pylori*
CI: Confidence interval
H2RA: H2-receptor antagonist
*Hp*: *Helicobacter pylori*
MALT: Mucosa-associated lymphoid tissue
NT: Northwest Territories
PCR: Polymerase chain reaction
PPI: Proton pump inhibitor
RNA: Ribonucleic acid
UBT: 13C-urea breath test
YT: Yukon
